# Smad7 in the hippocampus contributes to memory impairment in aged mice after anesthesia and surgery

**DOI:** 10.1186/s12974-023-02849-z

**Published:** 2023-07-28

**Authors:** Changliang Liu, Jiahui Wu, Ming Li, Rui Gao, Xueying Zhang, Shixin Ye-Lehmann, Jiangning Song, Tao Zhu, Chan Chen

**Affiliations:** 1 Department of Anesthesiology, West China Hospital, Sichuan University, Chengdu, China; 2 Laboratory of Anesthesia and Critical Care Medicine, National-Local Joint Engineering Research Center of Translational Medicine of Anesthesiology, West China Hospital, Sichuan University, Chengdu, China; 3 Diseases and Hormones of the Nervous System, University of Paris-Scalay Bicêtre Hosptial Bât. Grégory Pincus, 80 Rue du Gal Leclerc, Le Kremlin Bicêtre, 94276 CEDEX, Paris, France; 4 Monash Biomedicine Discovery Institute and Monash Data Futures Institute, Monash University, VIC Melbourne, Australia

**Keywords:** Postoperative cognitive dysfunction, Smad7, TGF-β, Inflammation, Apoptosis

## Abstract

**Background:**

Postoperative cognitive dysfunction (POCD) is a common neurological complication following anesthesia and surgery. Increasing evidence has demonstrated that neuroinflammation caused by systemic inflammatory responses during the perioperative period is a key factor in the occurrence of POCD. In addition, SMAD family member 7 (Smad7) has been confirmed to play vital roles in the pathogenesis and treatment of inflammatory diseases, such as inflammatory bowel disease. However, whether Smad7 participates in the regulatory process of neuroinflammation and apoptosis in the development of POCD is still unknown.

**Methods:**

In this study, a POCD mouse model was constructed by unilateral nephrectomy under anesthesia, and cognitive function was assessed using the fear conditioning test and open field test. The expression of Smad7 at the mRNA and protein levels in the hippocampus 3 days after surgery was examined by qRT-PCR, western blot and immunofluorescence assays. Furthermore, to identify whether the elevation of Smad7 in the hippocampus after unilateral nephrectomy contributes to cognitive impairment, the expression of Smad7 in the hippocampal CA1 region was downregulated by crossing Smad7^fl/fl^ conditional mutant mice and CaMKIIα-Cre line T29-1 transgenic mice or stereotaxic injection of shRNA–Smad7. Inflammation and apoptosis in the hippocampus were assessed by measuring the mRNA levels of typical inflammatory cytokines, including TNF-α, IL-1β, IL-6, CCL2, CXCL1, and CXCL2, and the protein levels of apoptotic proteins, including Bax and Bcl2. In addition, apoptosis in the hippocampus postoperation was investigated by a terminal deoxynucleotidyl transferase dUTP nick end labeling (TUNEL) staining assay. Finally, western blotting was used to explore how Smad7 mediates inflammation and apoptosis postoperation.

**Results:**

The results unequivocally revealed that elevated Smad7 in the hippocampal CA1 region significantly inhibited TGF-β signal transduction by blocking Smad2/3 phosphorylation, which enhanced neuroinflammation and apoptosis in the hippocampus and further led to learning and memory impairment after surgery.

**Conclusions:**

Our results revealed that Smad7 contributes to cognitive impairment after surgery by enhancing neuroinflammation and apoptosis in the hippocampus and might serve as a promising therapeutic target for the treatment of memory impairment after anesthesia surgery.

**Supplementary Information:**

The online version contains supplementary material available at 10.1186/s12974-023-02849-z.

## Introduction

Postoperative cognitive dysfunction (POCD) is one of the most common neurological complications in aged patients following anesthesia surgery [1]. It is often defined by an impairment of cognitive behaviors postsurgery, including trouble concentrating, learning and memory impairment, and executive dysfunction [2, 3]. Clinical evidence has demonstrated that approximately 10 ~ 60% of elderly patients suffer from POCD within the first week after surgery. Of these patients, one-third develop long-term dysfunction [4–6]. According to statistics, more than 200 million patients worldwide undergo anesthesia and surgery each year. With the increase in life expectancy, the number of elderly patients undergoing anesthesia and surgery will continue to increase [7]. POCD is usually associated with poor prognosis, including dysfunction in daily function, prolonged hospitalization and increased risk of mortality [3]. Therefore, the molecular mechanism of POCD urgently needs to be explored to identify potential therapeutic targets for clinical intervention. Recently, the term “perioperative neurocognitive disorders (PND)” was recommended as an overarching term for cognitive dysfunction occurring in the preoperative or postoperative period [8–13], which further reflects the urgency of exploring the pathogenesis of POCD. The development of POCD is closely associated with age, surgical duration, intraoperative infection and inflammation [14]. In addition, accumulating evidence has demonstrated that multiple pathological processes are involved in the development of POCD, including neuroinflammation [15] and neuronal apoptosis [16], oxidative stress [17], microglial activation [18], decreased blood brain barrier integrity [19], impairment of synaptic plasticity [20], overexpression and accumulation of β-amyloid protein [21], hyperphosphorylation of tau [22], etc. Although enormous efforts have been made in recent decades, the mechanism of POCD remains unclear.

Increasing research has demonstrated that neuroinflammation caused by systemic inflammatory responses following surgery is a key factor in the occurrence of POCD [15]. Persistent pain and anesthesia during the perioperative period may trigger microglial activation and lead to the release of inflammatory factors, including tumor necrosis factor α (TNF-α), interleukin 1β (IL-1β) and interleukin 6 (IL-6) [3, 18]. In addition, peripheral accompanying systemic inflammation induced by surgical trauma and continuous release of inflammatory factors have been proven to increase the inflammatory reaction in the central nervous system, resulting in the endogenous production and accumulation of proinflammatory cytokines [15, 23]. Recently, SMAD family member 7 (Smad7) has attracted considerable attention in inflammatory diseases, including inflammatory bowel diseases (IBDs), as an inhibitor of transforming growth factor β (TGF-β) [24]. Previous studies have shown that the specific antisense oligonucleotide of Smad7 could reduce the inflammatory responses in mice with IBDs through knockdown of Smad7 expression, which was also observed in patients with IBDs [24–26].

TGF-β is involved in many biological processes, including cell differentiation, proliferation, matrix protein synthesis, tissue repair, neoplasia and inflammatory disorders, as a multifunctional cytokine [27, 28]. Traditionally, TGF-β is an important anti-inflammatory factor that activates the TGF-β type I receptor by binding TGF-β type II receptor. Thereafter, the intracellular proteins Smad2/3 are phosphorylated by the activated TGF-β type I receptor and form a heteromeric complex with Smad4 to suppress the expression of inflammatory genes [28, 29]. TGF-β usually increases the expression of Samd7, which interacts with the TGF-β type I receptor and inhibits the phosphorylation of Smad2/3, blocking the signal transduction of TGF-β [27, 29, 30]. Consequently, immune cells produce increased levels of inflammatory cytokines and further exacerbate inflammation in many organs [29]. In addition, Smad7 has been reported to be associated with TGF-β-induced apoptosis in previous studies [31, 32]. For instance, elevated Smad7 could facilitate TGF-β-induced apoptosis in renal glomerular mesangial cells, which would be inhibited by the antisense oligonucleotide to Smad7 [32]. However, the underlying mechanism of Smad7 in regulating TGF-β-induced apoptosis is still unclear. Previous reports revealed that Smad7 might regulate TGF-β-induced apoptosis by interacting with proapoptotic molecules, such as the activation of p38 MAP kinase or nuclear factor κB (NF-κB) [31, 33]. Nevertheless, whether Smad7 participates in the regulatory process of neuroinflammation and apoptosis in the development of POCD is still unknown.

Therefore, the aim of the present work was to determine whether Smad7 increases neuroinflammatory responses and apoptosis in the central nervous system and further mediates the occurrence of cognitive impairment after anesthesia and surgery. In our study, unilateral nephrectomy under isoflurane anesthesia was performed to establish the cognitive dysfunction model induced by surgery in aged mice. The decreased freezing time of the mice in contextual test in the surgery group demonstrated significant learning and memory impairment. As reported in previous work, the hippocampal CA1 region plays critical roles in cognition, which results in dysfunction mediated by overactivated microglia and neuroinflammation [3, 34]. Interestingly, Smad7 overexpression was observed in the CA1 region of the hippocampus after unilateral nephrectomy. Overexpression of Smad7 triggered a severe neuroinflammatory reaction by blocking TGF-β signal transduction. Meanwhile, the elevated Smad7 in the hippocampus also enhanced neuronal apoptosis. The occurrence of neuroinflammation and apoptosis is heavily implicated in the development of cognitive decline after surgery. When the expression of Smad7 was knocked down by shRNA, neuroinflammation and apoptosis were significantly reduced and further attenuated cognitive decline postsurgery. The same results were observed in aged Smad7^−/−^ mice, further confirming that Smad7 might be a promising therapeutic target for the clinical invention of cognitive impairment induced by anesthesia surgery.

## Materials and methods

### Animals

Smad7^fl/fl^ conditional mutant mice (strain#: 017008) and CamKIIα-Cre line T29-1 transgenic mice (strain #: 005359) were purchased from Jackson Laboratories. Smad7 knockout mice in the CA1 pyramidal cell layer in the hippocampus were obtained by crossing Smad7^fl/fl^ conditional mutant mice and CaMKIIα-Cre line T29-1 transgenic mice [35]. Male mice homozygous for Smad7 knockout were screened through direct PCR analysis of tail DNA and maintained for 16 months for further experiments. Age-matched male wild-type C57BL/6 mice (16 ~ 18 months, 28 ~ 38 g) were purchased from Chengdu Dossy Experimental Animals Co., Ltd. (China). All mice were kept in a standard facility with a 12-h light–dark cycle, free access to food and water, ambient humidity of 20 ~ 50%, and temperature of 21 ~ 25 ℃.

All animal protocols in this work were approved by the Animal Care and Use Committee of Sichuan University and performed in strict compliance with the guidelines accepted by the National Institute of Health Guidelines for the Care and Use of Laboratory Animals. All efforts were made to minimize animal suffering and the number of animals used.

### Anesthesia and surgery

A mouse model of surgery-induced cognitive impairment was established through unilateral nephrectomy under anesthesia according to previous studies [36, 37]. Briefly, the mice were anesthetized by 3% isoflurane for induction, followed by 1.5% for maintenance. After that, a midline longitudinal incision under the costal margin was made, and the left kidney was removed. All the mice were administered 50 μL of 0.2% ropivacaine subcutaneously for postoperative analgesia and then kept on a heating pad to recover spontaneously. All surgical procedures were completed within 10 min to reduce the impact of isoflurane, which has been confirmed to affect neurocognition by regulating signaling pathways [38, 39]. Considering that anesthesia and surgery cannot be completely separated, the mice in the control group underwent neither anesthesia nor surgery.

### Fear conditioning test

Cognitive function was first assessed through the fear conditioning test, which has been confirmed to be a reliable strategy for investigating the learning and memory ability of rodents to associate a conditioned stimulus with an aversive, unconditioned stimulus [40, 41]. All mice were randomly divided into two groups and trained individually in a fear conditioning chamber (Ugo Basile, Italy) using a paradigm consisting of two pairs of conditional stimuli (tone, 75 dB, 5000 Hz, 20 s) and unconditional stimuli (foot shock, 0.75 mA, 2 s) on day 1 prior to the surgery. The total training time was set to 274 s. Animal motion speed was recorded by an infrared camera in front of the chamber. Thereafter, the mice were returned to the same chamber for testing on days 1, 3 and 7. For the contextual test, the mice were exposed to the chamber without any stimulation of tone and electric shock. After the mice rested for 90 min, the cue tone test was carried out in a context different from before. After a 180 s exploration period, the mice were given the same sound stimuli without an electric foot-shock stimulus and observed for 360 s. Thereafter, the percentage of freezing time in the contextual test and cued tone test were analyzed to express the memory of the mice after surgery.

### Open field test

The open field test was performed in a rectangular box (60 × 40 cm) with walls 20 cm in height in a quiet and dimly lit room to investigate the locomotor activity of the mice after surgery. Briefly, mice were transported to the testing room for 1 h before the open field test to acclimate to the surroundings. Afterwards, the mice were directly placed into the middle of the open field box and allowed to explore freely for 5 min. The total distance traveled in the box was automatically recorded and analyzed by a video-tracking system (Smart Version 2.5.20). Arenas were cleaned with 75% ethanol between each mouse.

### Stereotaxic injection of adeno-associated virus (AAV)

The AAV 2/9 vector interrupting the expression of Smad7 [pAKD–CMV–bGlobin–eGFP-H1–shRNA(Smad7), shRNA–Smad7] was designed and constructed by OBio Technology (Shanghai) Corp., Ltd. (China). In brief, 16-month-old wild-type mice were intraperitoneally injected with 1% sodium pentobarbital (50 mg/kg) and then restrained in a stereotaxic apparatus (RWD Life Science, China). Two microliters of shRNA–Samd7 (4.7 × 10^12^ vg/mL) or control AAV encoding GFP (vector) were injected into the bilateral hippocampal CA1 regions (anteroposterior, − 2.0 mm; mediolateral, ± 1.5 mm; dorsoventral, − 1.7 mm) at a rate of 0.5 μL/min through a microinjection pump. The body temperature of the mice was maintained using a homeothermic hating blanket during the procedure. After injection, the needle was kept in place for 5 min withdrawal, and all the mice were placed on a heating pad to recover spontaneously. The mouse model and behavioral tests were performed 3 weeks later.

### Western blotting

The mice were sacrificed on day 3 postoperation and the hippocampi were collected quickly on ice. The hippocampal tissues were homogenized using a hand-held homogenizer and lysed with lysis buffer supplemented with protease inhibitor cocktail and phenylmethylsulfonyl fluoride in an ice bath for 60 min. Total protein was collected by centrifugation at 13,000 rpm for 10 min at 4 ℃. The concentration of the protein was determined by a BCA relative protein quantification kit (Solarbio, China). Thereafter, ten micrograms of protein was loaded and separated by polyacrylamide gels before being transferred to poly(vinylidene difluoride) membranes with a pore diameter of 0.22 μm (Millipore, Bedford, MA, USA). The membranes were blocked with 5% nonfat milk (BD bioscience) in Tris-buffered saline with 0.1% Tween-20 (TBST) for 1 h at room temperature and then incubated with primary antibodies: rabbit anti-Smad7 polyclonal antibody (Proteintech, 25840-1-AP), rabbit anti-Bax polyclonal antibody (Proteintech, 50599-2-Ig), rabbit anti-Bcl2 polyclonal antibody (Proteintech, 26,593–1-AP), rabbit anti-Smad2/3 monoclonal antibody (Abcam, ab232326), rabbit anti-Smad4 polyclonal antibody (Proteintech, 17387-1-AP), and rabbit anti-p-Smad2/3 polyclonal antibody (Abcam, ab272332). Thereafter, the membranes were washed with TBST and incubated with horseradish peroxidase-conjugated secondary antibodies for another 1 h at room temperature in TBST containing 5% nonfat milk. The blots were developed using an Enhanced Chemiluminescence Kit (Thermo Pierce, Waltham, MA, USA) and visualized on a chemiluminescence image analysis system (Amersham Imager 600). The gray value of the targeted protein band intensity was quantified using ImageJ software.

### Quantitative real-time polymerase chain reaction (qRT-PCR)

Total RNA from the mouse hippocampus was extracted through the TRIzol method. RNA concentration was determined by spectrophotometry using a Nanodrop ND-1000 (Thermo Fisher Scientific), and equal amounts of RNA were used for cDNA synthesis. cDNA was reverse-transcribed using a PrimeScript RT Reagent Kit with gDNA Eraser (Vazyme, China). Thereafter, templates were amplified by real-time polymerase chain reaction using primers for the mouse gene listed in Table 1. Each sample was run in sextuplicate at least in a 20 μL reaction with 250 nM forward and reverse primers, 10 μL of SYBR Green Supermix and 20 ng of cDNA. Polymerase chain reactions were performed with an initial 2 min incubation at 95 ℃, followed by 40 cycles at 95 ℃ for 10 s, 55 ℃ for 10 s, and 60 ℃ for 20 s in a Real-Time PCR system (Bio-Rad, USA). The relative quantification of the sample transcripts was calculated using the ^ΔΔ^Cq method with 18S as a control.

**Table 1:** List of polymerase chain reaction primers for real-time qRT-PCR analysis

Gene	Forward (5ʹ-3ʹ)	Reverse (5ʹ-3ʹ)
CXCL1	GCACCCAAACCGAAGTCA	AAGCCAGCGTTCACCAGA
CXCL2	GCCCAGACAGAAGTCATAGC	AGCGAGGCACATCAGGTA
CCL2	CCCCAAGAAGGAATGGGTCC	GTGCTGAAGACCTTAGGGCA
IL-1β	TGCCACCTTTTGACAGTGATG	CATCTCGGAGCCTGTAGTGC
IL-6	TGAGAAAAGAGTTGTGCAATGG	GGAGAGCATTGGAAATTGGGG
TNF-α	CTGTGAAGGGAATGGGTGTT	CAGGGAAGAATCTGGAAAGGTC
Smad7	CAAACCAACTGCAGGCTGTC	TGAACTCGTGGTCATTGGGC
18s	TTGACTCAACACGGGAAACC	AGACAAATCGCTCCACCAAC

### Immunofluorescence imaging

The mice were deeply anesthetized with sodium pentobarbital via intraperitoneal injection at a dose of 60 mg/kg. Afterwards, the mice were perfused with cold PBS for 5 min, followed by 4% paraformaldehyde solution for 15 min. Then, the brains were excised and postfixed overnight in 4% paraformaldehyde at 4 ℃ and dehydrated in 30% sucrose–PBS. The brains were mounted in optimal cutting temperature embedding medium, frozen and cut coronally at a 40 μm thickness on a cryostat. Next, the brain slices were fixed with 4% paraformaldehyde for another 10 min and incubated with 0.3% Triton X-100 for 10 min, followed by blocking with 10% normal donkey serum containing 0.1% Triton X-100 for 60 min at room temperature. Afterwards, the brain sections were incubated overnight at 4 ℃ with the following primary antibodies: mouse anti-NeuN monoclonal antibody (Proteintech, 66836-1-Ig), mouse anti-Iba1 monoclonal antibody (Abcam, ab283319) and rabbit anti-Smad7 polyclonal antibody (Proteintech, 25840-1-AP). After being washed three times in PBS, the brain sections were incubated with secondary antibodies conjugated with Alexa 488 or 555 for 60 min at room temperature. The slices were mounted with Antifade Mounting Medium containing DAPI (Beyotime, China) and observed using a laser scanning confocal microscope (Leica CM DMI8).

### TUNEL staining

A terminal deoxynucleotidyl transferase dUTP nick end labeling (TUNEL) staining assay was performed to investigate apoptosis in the hippocampus after surgery using an in situ cell death detection kit (Roche) according to the manufacturer’s instructions. Briefly, the brains were collected at day 3 postoperation after transcardiac perfusion with PBS for 5 min and 4% paraformaldehyde for 15 min. Then, the tissues were fixed overnight and dehydrated in 30% sucrose for 48 h. Thereafter, the tissues were dissected into 10 μm thick sections and stained with the TUNEL staining kit. Nuclei were stained with DAPI and sealed with anti-fluorescence quenching sealing solution. Apoptotic cells were imaged using fluorescence microscopy at excitation wavelengths of 405 nm and 488 nm.

### Statistical analysis

Quantitative data are displayed as the means ± standard deviations and were analyzed by ANOVA. All experiments were carried out at least in triplicate. Student’s *t* test was utilized as a post hoc test. *P* < 0.05 was considered statistically significant.

## Results

### Unilateral nephrectomy led to cognitive decline

**Fig. 1:**
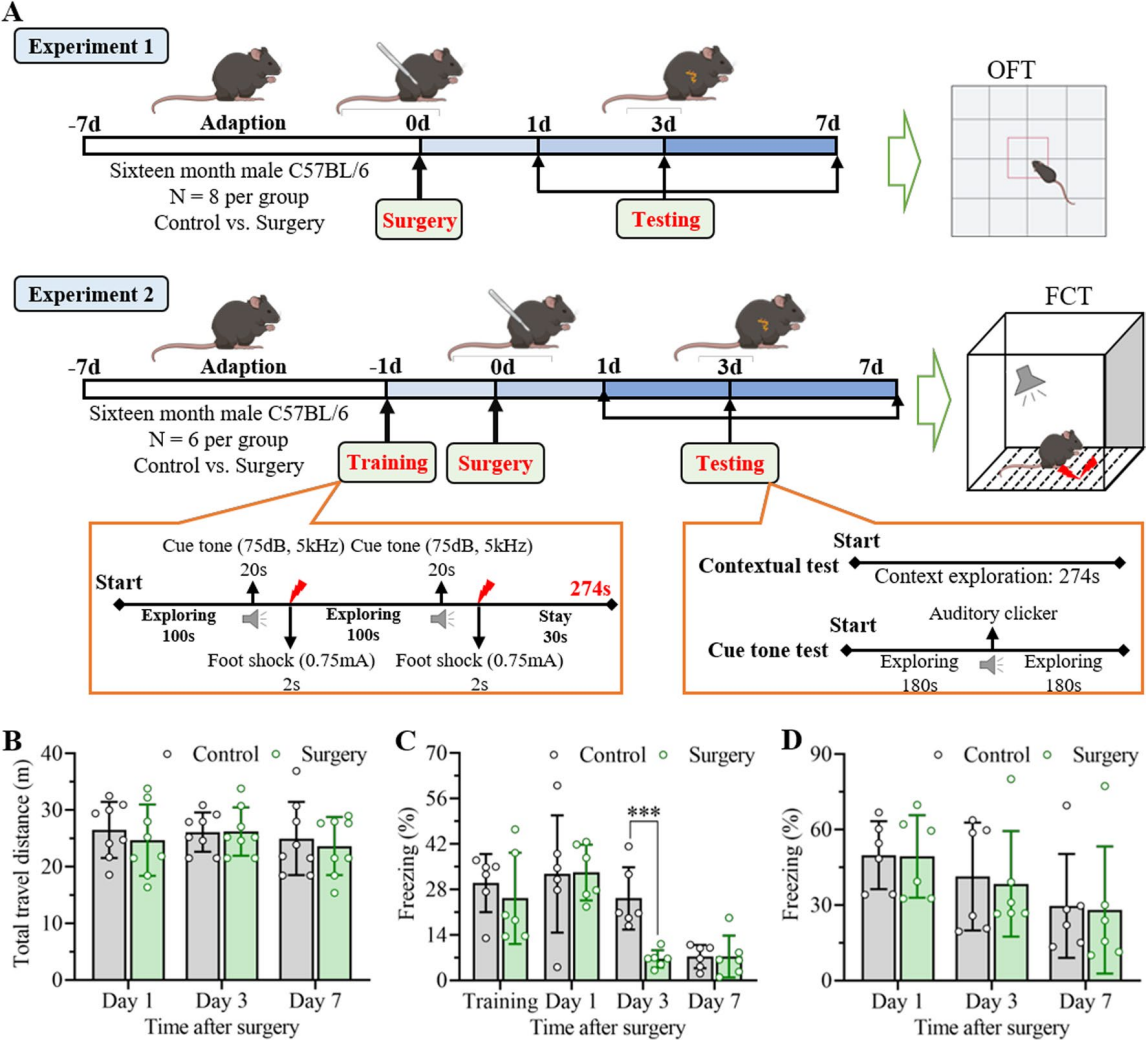
Unilateral nephrectomy under anesthesia induced significant cognitive decline in aged mice. **A** Flowchart diagrams display the timeline of experimental procedures in this study. In experiment 1, unilateral nephrectomy under anesthesia was performed in 16-month-old male mice after at least 7 days of adaptation. Then, the mice were used to conduct open field tests on days 1, 3 and 7 after surgery. Age-matched mice without anesthetic operation were used as controls. In experiment 2, the mice were allowed to adapt to the environment for at least 7 days and trained in a fear conditioning chamber on day 1 before surgery. Thereafter, the mice were administered FCT on days 1, 3 and 7 after unilateral nephrectomy. **B** Open field test was performed on days 1, 3 and 7 after unilateral nephrectomy to evaluate the effects of anesthesia and surgery on aged mice. The data are presented as the mean ± standard error (*n* = 8). **C** Percent freezing time in the contextual test was determined at days 1, 3 and 7 after the operation to examine contextual fear memory. The percent freezing time during the training session at 1 day preoperation was used as the baseline cognitive function. The data are presented as the mean ± standard error (*n* = 6). ***P < 0.001. **D** Percent freezing time in the cue tone test was detected on days 1, 3 and 7 after surgery to examine auditory cue fear memory. The data are presented as the mean ± standard error (*n* = 6)

In the present work, we established a cognitive dysfunction mouse model through unilateral nephrectomy under isoflurane anesthesia. The mice in the control group underwent neither anesthesia nor surgery due to the indivisibility of anesthesia and surgery. Thereafter, longitudinal neurobehavioral tests were performed to evaluate the learning and memory abilities of the mice postoperation. All the experiments are displayed in the flowcharts (Fig. 1A).

The open field test was initially administered on days 1, 3 and 7 to assess the effects of anesthesia and surgery on locomotor activity. The total travel distance during the 5 min exploration in the open field chamber displayed no significant differences between the control and surgery groups, demonstrating negligible influences of the surgical process on locomotor activity (Fig. 1B). Afterwards, cognitive function was determined through fear conditioning tests on days 1, 3 and 7 after surgery, in which environmental correlation tests were used to evaluate contextual fear memory, while cue tone tests were used to assess auditory cue fear memory. As shown in Fig. 1C, no significant differences in freezing time were observed in the training phase, revealing equal baseline learning and memory abilities in the control and surgery groups. Interestingly, the freezing time in the contextual test decreased conspicuously on day 3 after unilateral nephrectomy. Nevertheless, no differences in freezing time in the cue tone test between the surgery and control groups were detected at any timepoint (Fig. 1D). These results demonstrated that unilateral nephrectomy under anesthesia induced remarkable learning and memory impairment.

### Smad7 is upregulated in hippocampal neurons after unilateral nephrectomy

**Fig. 2:**
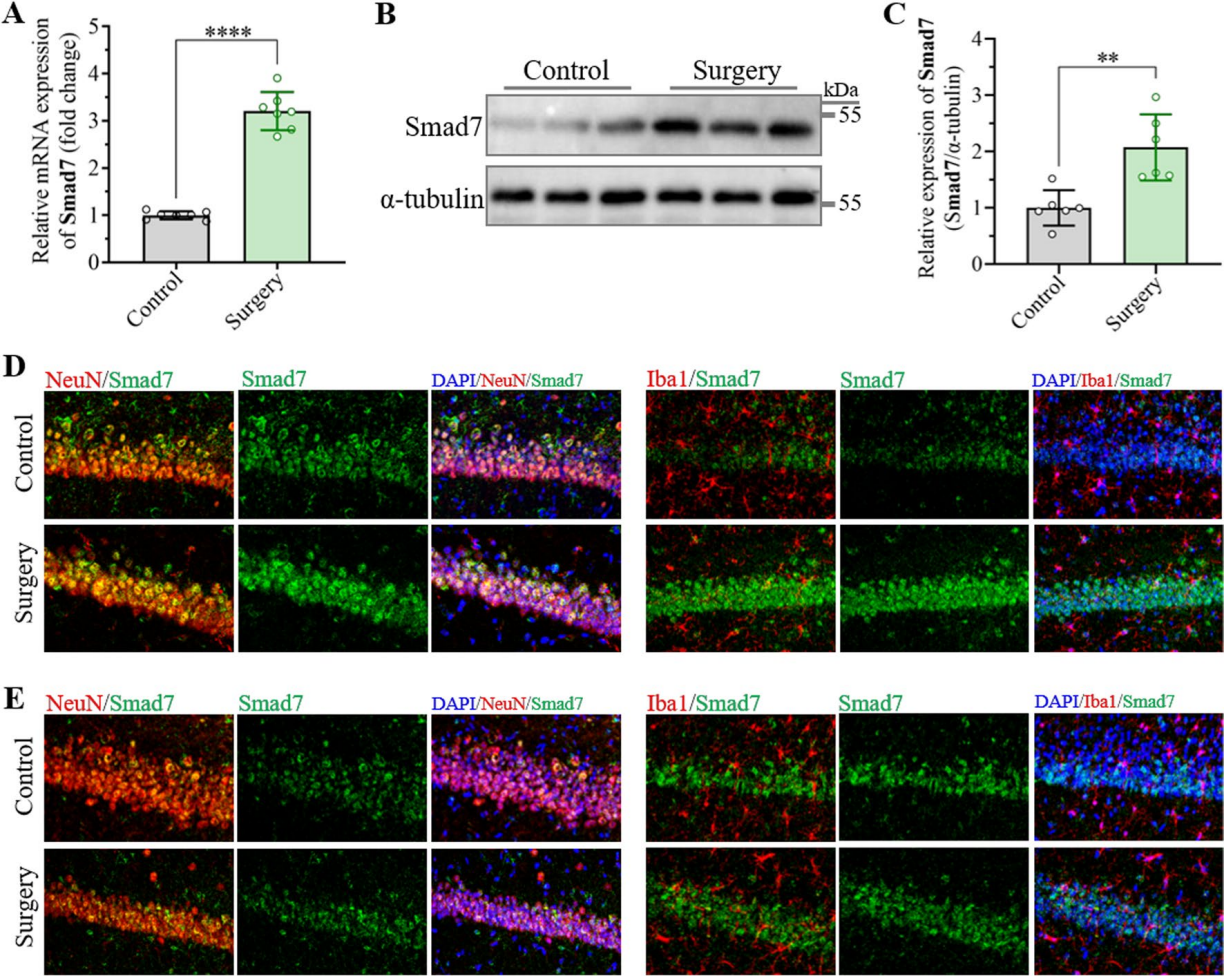
Unilateral nephrectomy under anesthesia induced upregulation of Smad7 expression in neurons of the hippocampal CA1 region. **A** Expression of Smad7 at the mRNA level in the hippocampus was measured by qRT-PCR assay at day 3 after surgery. The data are presented as the mean ± standard error (*n* = 7). ****P < 0.0001. **B** Representative western blots of Smad7 in the hippocampus at day 3 after surgery. **C** Expression of Smad7 protein in the hippocampus was determined by western blotting assay on day 3 after unilateral nephrectomy. The data are presented as the mean ± standard error (*n* = 6). **P < 0.01. **D**, **E**. Representative confocal images of Smad7 expression in neurons (NeuN) and microglia (Iba1) in the (**D**) CA1 region and (**E**) CA2/3 region of the hippocampus

The expression of Smad7 at the mRNA level was first examined in the hippocampus 3 days after unilateral nephrectomy under anesthesia through qRT-PCR. The results showed that the mRNA levels of Smad7 in the hippocampus from the surgery group were significantly higher than those in the control group (Fig. 2A). Western blot assays were further performed to determine the protein expression of Smad7 in the hippocampus, which displayed significant upregulation in the hippocampus after unilateral nephrectomy (Fig. 2B, C). Thereafter, dual immunofluorescence staining of Samd7 combined with the marker for neurons (NeuN) or microglia (Iba1) was performed to determine Smad7 expression in different cell types and hippocampal regions. Bright fluorescent signals of Smad7 were observed in the CA1 region of the hippocampus in the surgery group compared with the control group, which revealed that upregulated Smad7 was mainly located in the CA1 region of the hippocampus. In addition, immunofluorescence staining showed that Smad7 was highly expressed in neurons and to a lesser extent in microglia (Fig. 2D, E, Additional file 1: Fig. S1). In addition, we detected Smad7 expression in the prefrontal cortex and amygdala to examine whether Smad7 from other brain regions is involved in the occurrence of POCD. The results showed no significant difference in the amygdala in the surgical group mice compared to the control group. However, the expression of Smad7 in the prefrontal cortex was decreased significantly (Additional file 1: Fig. S2). Taken together, these results suggested that the increased expression of Smad7 in hippocampal neurons might play a vital role in the development of cognitive decline induced by anesthesia surgery.

### Inhibition of Smad7 expression in the hippocampus attenuated unilateral nephrectomy-induced cognitive decline

**Fig. 3:**
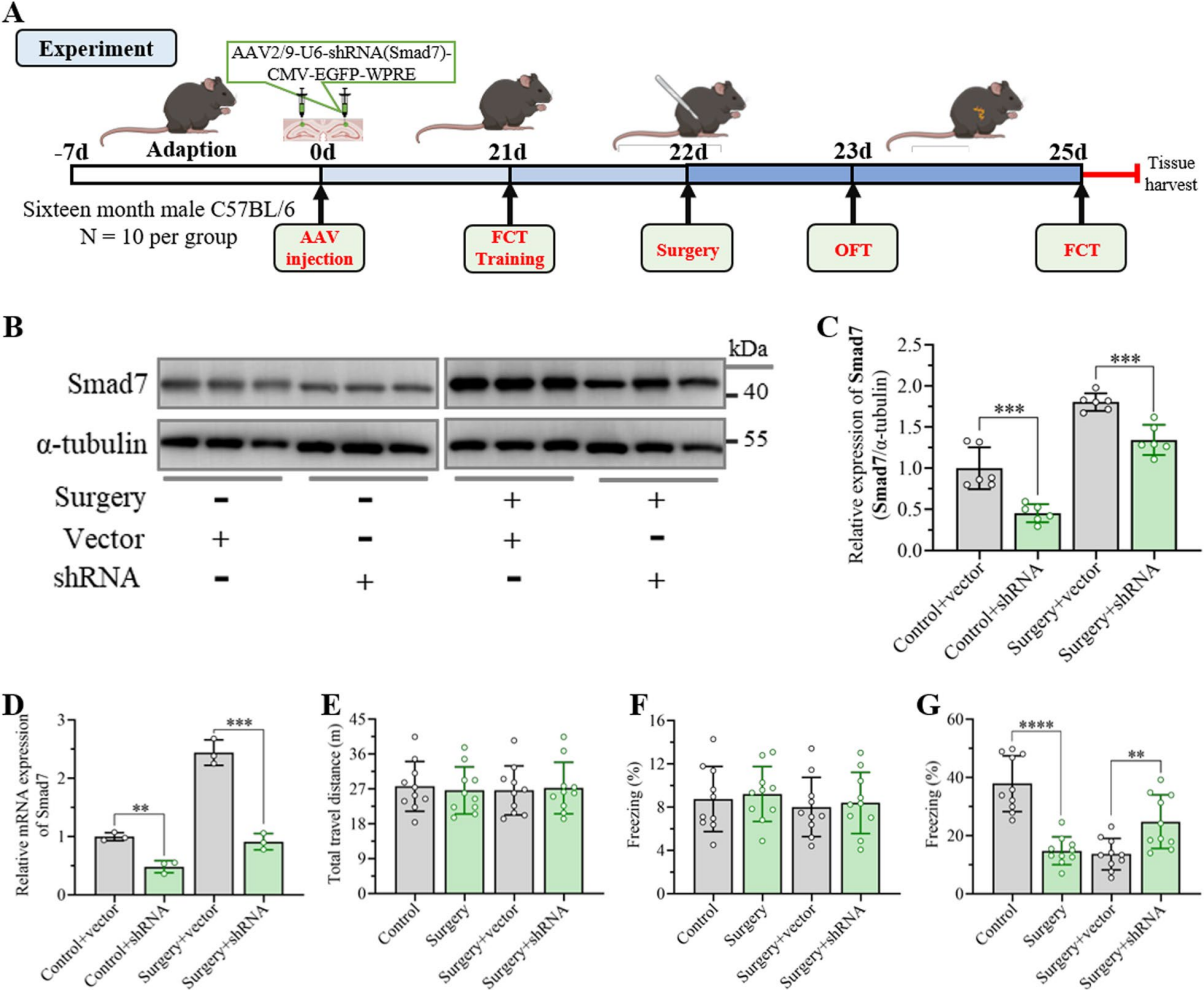
Knockdown of Smad7 in the hippocampus attenuated cognitive deficits induced by unilateral nephrectomy. **A** Timeline of the experimental procedure used in this study. Unilateral nephrectomy was conducted after AAV injection for 21 days, and then the open field test and fear conditioning test were performed to evaluate locomotor activity and cognitive function on day 1 and day 3 after surgery, respectively. **B** Representative western blots of Smad7 in the hippocampus at day 21 after AAV injection. **C** Quantification of Smad7 expression in the hippocampus according to western blots. The data are presented as the mean ± standard error (*n* = 6). ***P < 0.001. **D** Expression of Smad7 at the mRNA level was measured by qRT-PCR. The data are presented as the mean ± standard error (*n* = 3). **P < 0.01, ***P < 0.001. **E** Open field test was performed to examine the effects of AAV injection and surgery on locomotor activity. The data are presented as the mean ± standard error (*n* = 10). **F** Percent freezing time during the training session at day 1 preoperation was detected to examine the cognitive baseline. The data are presented as the mean ± standard error (*n* = 10). **G** Percent freezing time was determined at day 3 postoperatively to examine contextual fear memory. The data are presented as the mean ± standard error (*n* = 10). **P < 0.01, ****P < 0.0001

To identify whether the elevation of Smad7 in neurons in the hippocampal CA1 region after unilateral nephrectomy contributed to cognitive impairment, a serotype 2/9 adeno-associated virus encoding shRNA of Smad7 (shRNA–Smad7) was constructed and injected into the bilateral hippocampus to inhibit Smad7 expression. The expression of Smad7 in the hippocampus was detected by western blot assay at 21 day postinjection. The results showed that shRNA–Smad7 significantly inhibited the expression of Smad7 in the hippocampus in both the control and surgery groups (Fig. 3B, C). qRT-PCR assays further confirmed the inhibition of Smad7 expression by shRNA–Smad7 at the mRNA level, suggesting the excellent silencing effect of shRNA–Smad7 in the hippocampus (Fig. 3D). Thereafter, an open field test and fear conditioning test were performed to examine the effects of Smad7 knockdown in the hippocampus on cognitive behavior in aged mice after anesthesia surgery, as shown in the flowcharts of Fig. 3A. Open field tests were first carried out on day 1 postoperation to investigate the effect of AAV injection and surgery on locomotor function. Predictably, there were no differences in the total travel distance of the control and surgery groups, reflecting the slight influences on the locomotor ability of aged mice (Fig. 3E). Next, in the fear conditioning test, the percentage of freezing time preoperation showed no differences in the AAV vector injection group and shRNA–Smad7 injection group compared to the non-AAV injection groups, demonstrating that AAV injection had no adverse effects on cognitive behaviors (Fig. 3F). The mice without any AAV injection exhibited a significant decline in the percentage of freezing time at day 3 postsurgery compared with the control group, revealing a significant cognitive deficit induced by anesthesia and surgery. Meanwhile, the mice treated with AAV vector exhibited no differences with the surgery groups, demonstrating that the AAV vector has no effects on improving cognition. Interestingly, the mice treated with shRNA–Smad7 preoperation exhibited longer freezing times than the mice treated with AAV vector or without treatment after unilateral nephrectomy, suggesting the involvement of Smad7 in the development of cognitive impairment induced by anesthesia and surgery (Fig. 3G).

**Fig. 4:**
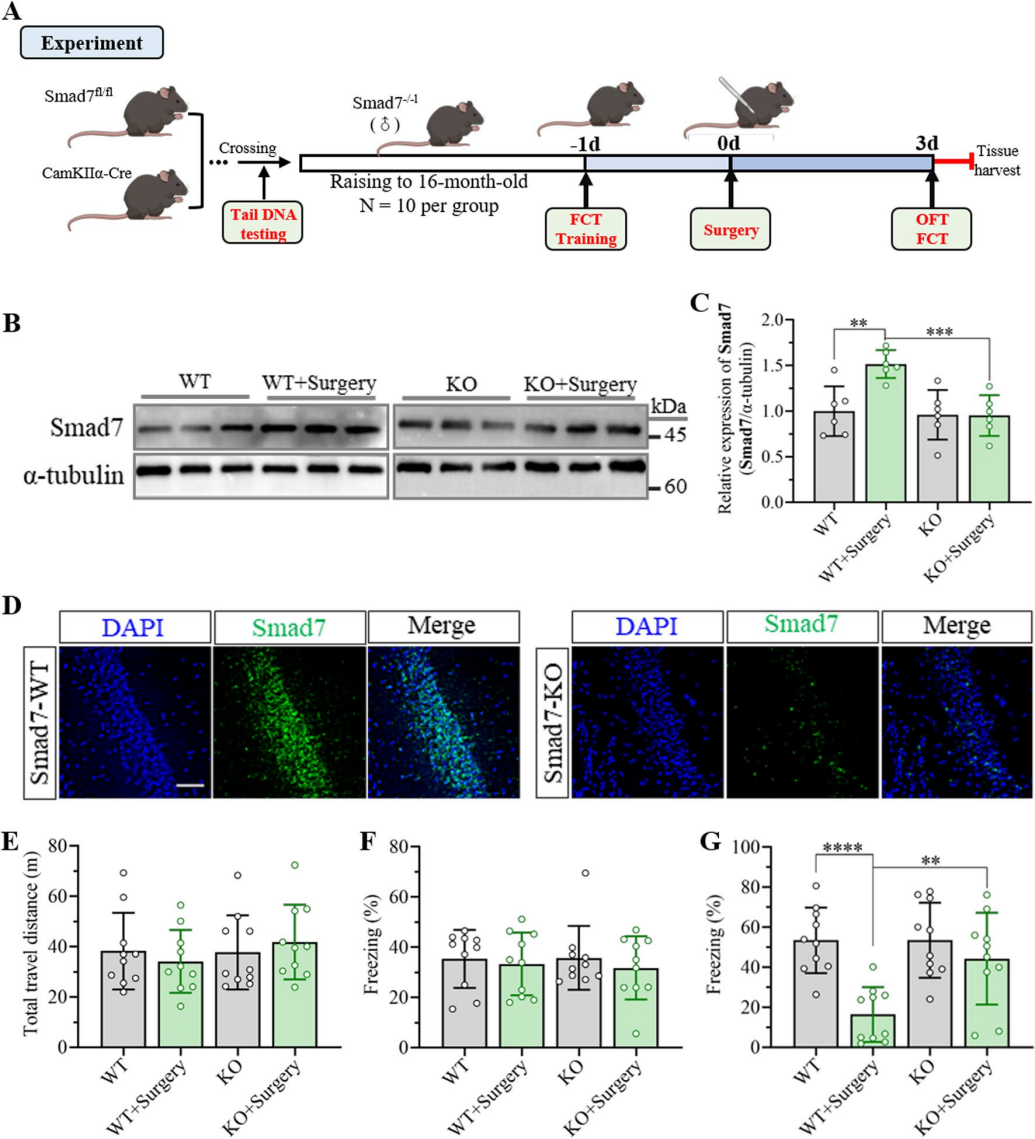
Smad7 knockout in the hippocampal CA1 region improves cognitive decline after unilateral nephrectomy. **A** Timeline of the experimental procedure used in this study. Male mice homozygous for Smad7 knockout were obtained by crossing Smad7^fl/fl^ conditional mutant mice and CaMKIIα-Cre line T29-1 transgenic mice. Then, the mice were fed to 16 months for further experiments. OFT and FCT were performed on day 3 after unilateral nephrectomy. **B** Representative western blots of Smad7 in the hippocampus. **C** Quantification of Smad7 expression in the hippocampus according to western blots. The data are presented as the mean ± standard error (*n* = 6). **P < 0.01, ***P < 0.001. **D** Expression of Smad7 in the hippocampal CA1 region was further detected by immunofluorescence assay. Scale bar: 50 μm. **E** Open field test was performed to examine the effects of Smad7 knockout and surgery on locomotor activity. The data are presented as the mean ± standard error (*n* = 10). **F** Percent freezing time during the training session at day 1 preoperation was detected to examine the cognitive baseline. The data are presented as the mean ± standard error (*n* = 10). **G** Percent freezing time was determined at day 3 postoperation to examine contextual fear memory. The data are presented as the mean ± standard error (*n* = 10). **P < 0.01, ****P < 0.0001

Furthermore, Samd7 knockout (Smad7^−/−^) mice in the hippocampal CA1 region were constructed by crossing Smad7^fl/fl^ conditional mutant mice and CaMKIIα-Cre line T29-1 transgenic mice to investigate the effects of Smad7 on the development of cognitive dysfunction following anesthesia and surgery. Smad7^fl/fl^ mutant mice possess LoxP sites flanking exon 1 of the Smad7 gene, which will assign exon 1 on offspring in Cre-expressing tissue when these mutant mice are bred to mice expressing Cre recombinase. CaMKIIα-Cre transgenic mice from line T29-1 (Tg(Camk2a-cre)T29-1Stl) can direct the expression of Cre recombinase through the CaMKIIα promotor. Cre-mediated recombination occurs in the pyramidal cell layer after crossing with a strain containing the Loxp site flanked sequence of interest. Previous research has confirmed that crossing the CaMKIIα-Cre line T29-1 mice with a Cre-dependent lacZ reporter line resulted in Cre recombinase expression in the forebrain, predominantly in the CA1 pyramidal cell layer in the hippocampus during the third to fourth postnatal week [35]. Direct PCR analysis of tail DNA revealed that the Smad7 gene in the hippocampus was successfully knocked out (Additional file 1: Fig. S3). Thereafter, the expression of Samd7 in the hippocampus was examined through western blotting, which indicated the significant knockout of Smad7 in the hippocampus in both the control and surgery groups (Fig. 4B, C). Immunofluorescence images further showed that the expression of Smad7 in the hippocampal CA1 region was effectively knocked out (Fig. 4D). Thereafter, an open field test and fear conditioning test were carried out to evaluate cognitive behavior following surgery when the mice were 16 months according to the experimental timeline shown in Fig. 4A. In the open field test, there were no differences in locomotor function between wild-type mice and Smad7^−/−^ mice after unilateral nephrectomy for 3 days (Fig. 4E). In the fear conditional test, the percentage of freezing time exhibited no difference before surgery, suggesting that Smad7 knockout in the hippocampal CA1 region had no effect on memory behavior (Fig. 4F). Importantly, the wild-type mice exhibited remarkable cognitive impairment according to the significant decrease in freezing time in Fig. 4G. However, the Smad7 knockout group showed a higher percentage of freezing time than the wild-type group after unilateral nephrectomy, suggesting that Smad7 knockout attenuated cognitive dysfunction induced by anesthesia surgery.

### Inhibition of Smad7 expression in the hippocampus attenuates the inflammatory response induced by anesthesia and surgery

**Fig. 5:**
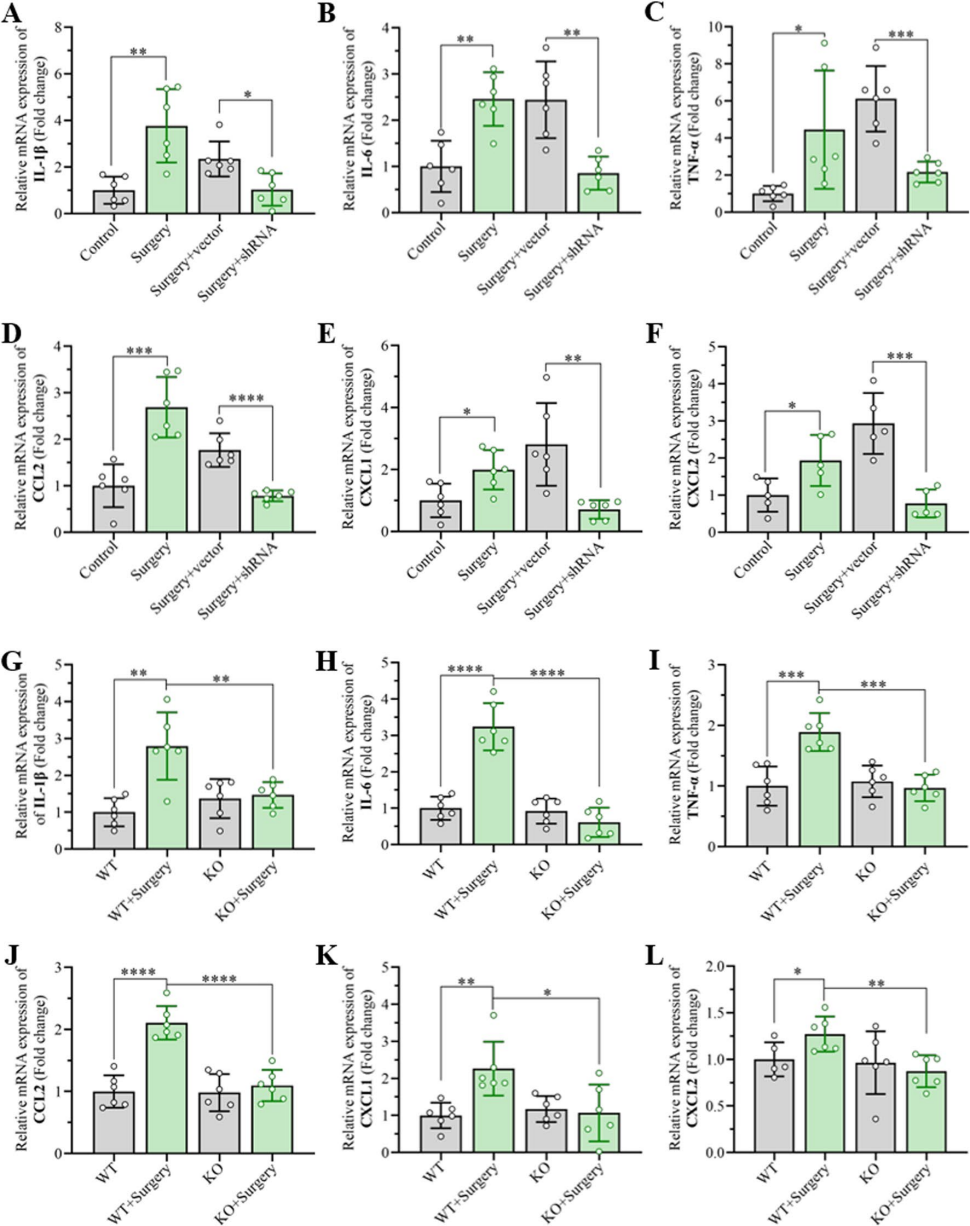
Inhibition of Smad7 expression in the hippocampus improved the inflammatory response induced by unilateral nephrectomy. **A**–**F** Expression of inflammatory cytokines in the hippocampus at the mRNA level, including (**A**) IL-1β, (**B**) IL-6, (**C**) TNF-α, (**D**) CCL2, (**E**) CXCL1 and (**F**) CXCL2, was examined by qRT-PCR. Unilateral nephrectomy was performed at day 21 post AAV injection, and hippocampal tissues were harvested at day 3 after surgery. **G**–**L** mRNA expression of inflammatory cytokines in the hippocampus, including (**G**) IL-1β, (**H**) IL-6, (**I**) TNF-α, (**J**) CCL2, (**K**) CXCL1 and (**L**) CXCL2, was examined by qRT-PCR in Smad7^−/−^ mice after anesthesia and surgery. Unilateral nephrectomy was performed when the Samd7^−/−^ mice were 16 months, and hippocampal tissues were harvested 3 days after surgery. The data are presented as the mean ± standard error (*n* = 6). *P < 0.05, **P < 0.01, ***P < 0.001, ****P < 0.0001

To pinpoint the potential role of upregulated Smad7 in the hippocampus on surgery-triggered cognitive deficit, the levels of proinflammatory cytokines, including IL-1β, IL-6 and TNF-α, at the mRNA level, were investigated at day 3 postoperation through qRT-PCR. The results showed that these proinflammatory cytokines increased significantly in the hippocampus of the surgery group compared with the control group. AAV vector injection in the hippocampus exhibited no positive effects on improving the inflammatory response. Interestingly, the expression of these proinflammatory cytokines was inhibited remarkably by shRNA–Smad7, suggesting that the elevated Smad7 in the hippocampus after unilateral nephrectomy plays important roles in the inflammatory response (Fig. 5A–C). Meanwhile, we also found that the expression of several chemokines, including CCL2, CXCL1 and CXCL2, increased significantly in the hippocampus at day 3 after unilateral nephrectomy. However, the expression of these chemokines decreased dramatically when Smad7 expression was silenced by shRNA–Smad7 (Fig. 5D–F). Furthermore, the inflammatory responses in the hippocampus mediated by Smad7 after unilateral nephrectomy were examined using Smad7^−/−^ mice. The results showed that the expression of the inflammatory cytokines mentioned above was significantly elevated at the mRNA level, suggesting an increase in inflammatory responses in the hippocampus induced by anesthesia and surgery. These inflammatory cytokines were significantly reduced in the hippocampus of Smad7^−/−^ mice (Fig. 5G–L). Taken together, these results indicated that the elevated Smad7 in the hippocampus in aged mice after anesthesia and surgery might mediate cognitive dysfunction by enhancing neuroinflammatory responses, which could be reduced by inhibiting the expression of Smad7 in the hippocampus.

### Inhibition of Smad7 expression in the hippocampus decreased apoptosis after unilateral nephrectomy

**Fig. 6:**
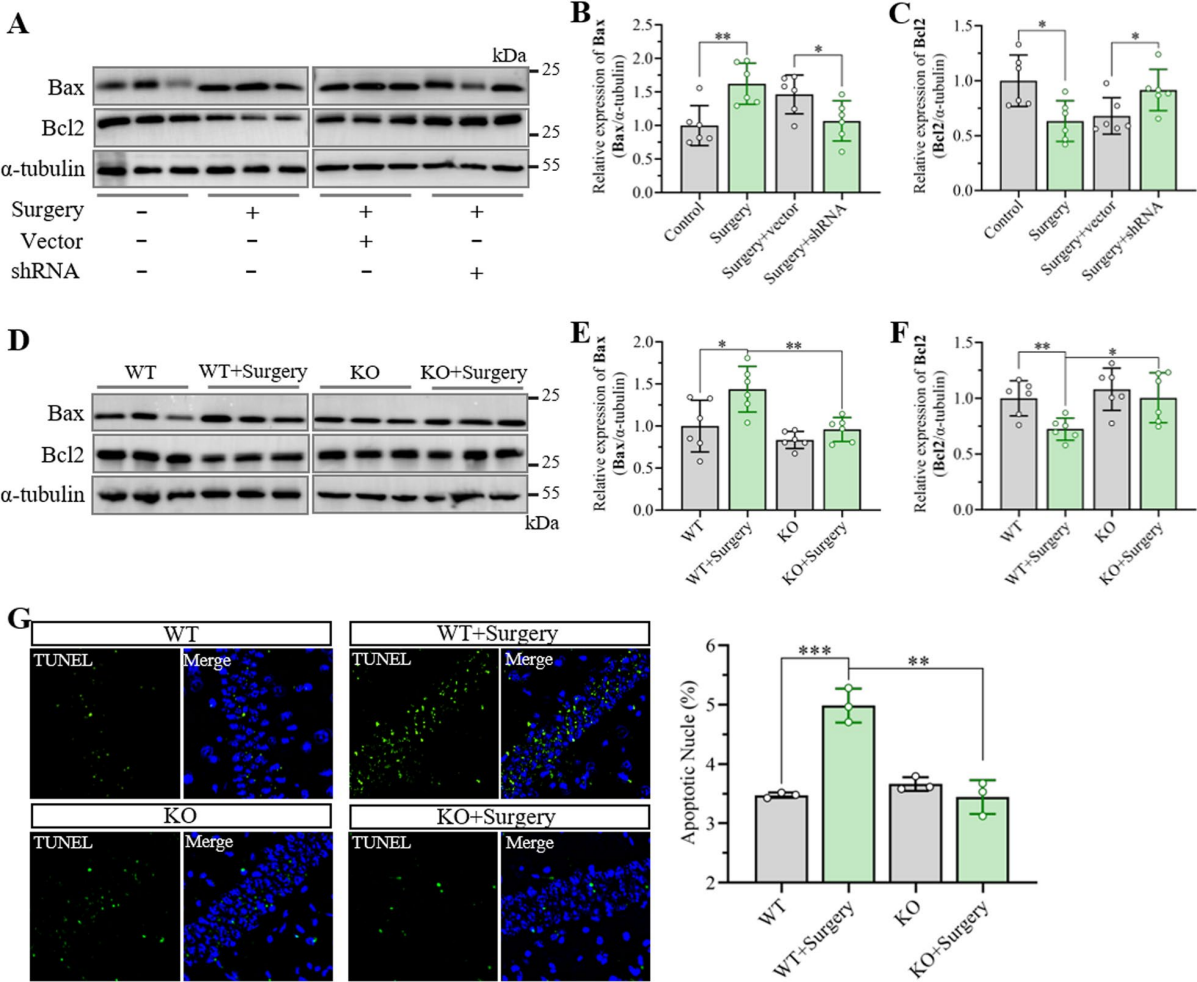
Inhibition of Smad7 expression in the hippocampus attenuated apoptosis induced by unilateral nephrectomy. **A** Representative western blots of Bax and Bcl2 in the hippocampus. The mice underwent unilateral nephrectomy at day 21 post AAV injection, and the hippocampal tissues were harvested at day 3 after surgery. **B**, **C** Quantification of (**B**) Bax and (**C**) Bcl2 expression in the hippocampus according to western blots. The data are presented as the mean ± standard error (*n* = 6). *P < 0.05, **P < 0.01. **D** Representative western blots of Bax and Bcl2 in the hippocampus. Unilateral nephrectomy was performed when the Samd7.^−/−^ mice were 16 months, and the hippocampi were harvested at day 3 after surgery. **E**, **F** Quantification of (**E**) Bax and (**F**) Bcl2 expression in the hippocampus according to western blots. The data are presented as the mean ± standard error (*n* = 6). *P < 0.05, **P < 0.01. **G** Representative images of TUNEL staining in the hippocampus at day 3 after unilateral nephrectomy and the quantitation of TUNEL staining. The data are presented as the mean ± standard error (*n* = 6). **P < 0.01, ***P < 0.01

Previous studies have confirmed that Bax is an important proapoptotic protein, that can trigger apoptosis by inducing permeation of the outer membrane of mitochondria [42]. In contrast, Bcl2 protein plays a vital role in anti-apoptosis by inhibiting the development of oxidative stress, decreasing the release of pro-apoptotic cytokines from mitochondria, inhibiting the function of Bax and Caspases, and maintaining cellular calcium homeostasis [43]. Therefore, the expression of Bax and Bcl2 was determined by western blotting assay, which showed that Bax increased significantly in the hippocampus in the surgery group with/without AAV vector treatment after unilateral nephrectomy for 3 days. The expression of Bcl2 was dramatically downregulated compared with that in the control mice. Interestingly, when the expression of Smad7 was inhibited by shRNA–Smad7, the expression of the above alterations was significantly altered (Fig. 6A–C). Meanwhile, the expression of Bax and Bcl2 protein in the hippocampus was further examined in aged Smad7^−/−^ mice. The results showed that proapoptotic cytokines increased remarkably in the hippocampus in wild-type mice after surgery, while antiapoptotic cytokines decreased. In contrast, the expression of Bax and Bcl2 in the hippocampus was reversed dramatically in Smad7^−/−^ mice after unilateral nephrectomy (Fig. 6D–F). Next, TUNEL staining was performed to detect apoptosis in the hippocampus after anesthesia and surgery. The increased green fluorescent signal in the hippocampus after surgery in the wild-type mice demonstrated enhanced cell apoptosis, whereas hippocampal apoptosis was significantly reduced in the Smad7^−/−^ mice after surgery. Thus, these results indicated that Smad7-mediated elevation of hippocampal apoptosis also plays a critical role in cognitive decline.

### Smad7 mediates cognitive impairment after unilateral nephrectomy by inhibiting Smad2/3 phosphorylation

**Fig. 7:**
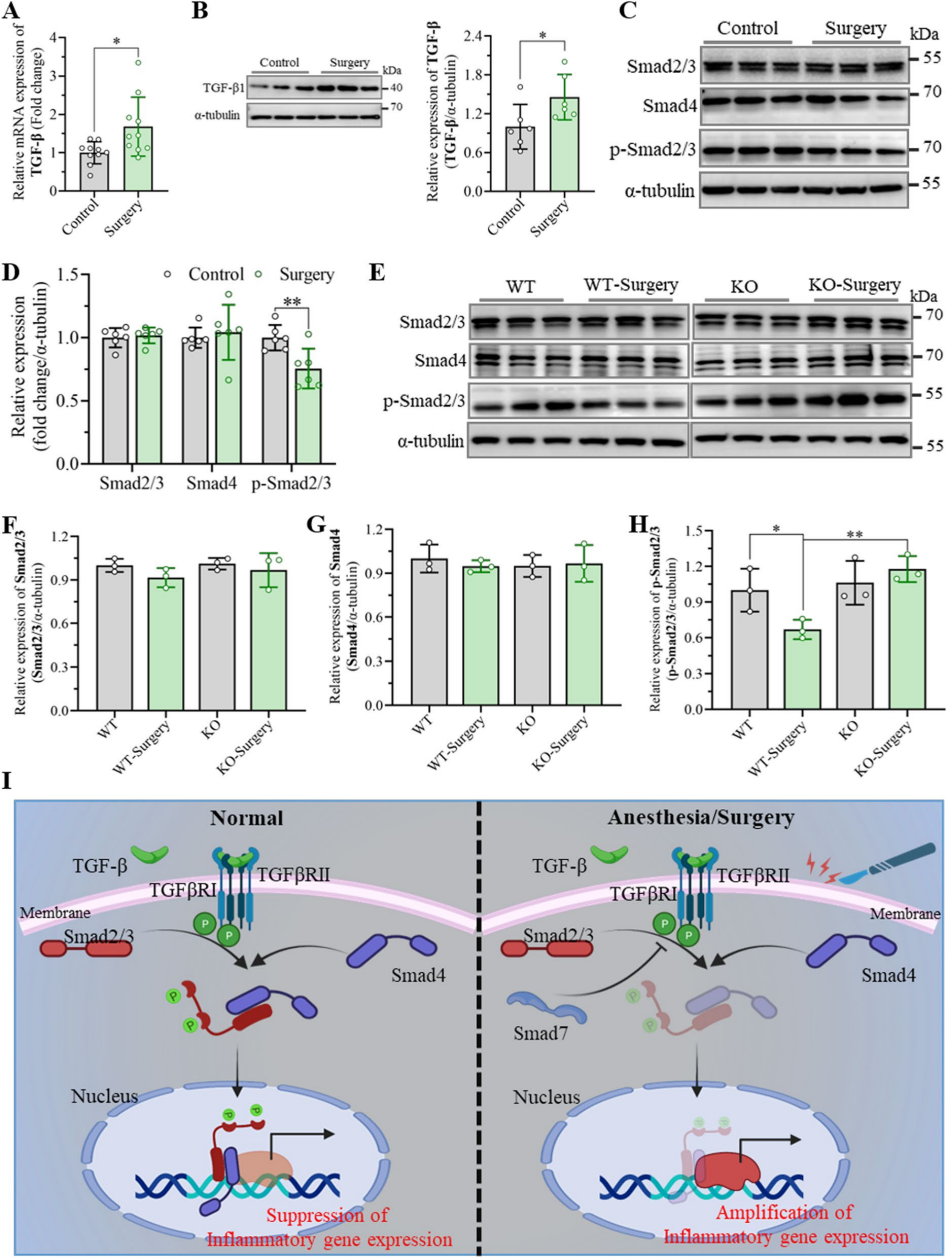
Smad7 mediates cognitive dysfunction after unilateral nephrectomy by inhibiting Smad2/3 phosphorylation. **A** Expression of TGF-β at the mRNA level in the hippocampus was measured by qRT-PCR assay at day 3 after surgery. The data are presented as the mean ± standard error (*n* = 10). *P < 0.05. **B** Representative western blots and quantification of Smad7 in the hippocampus at day 3 after surgery. The data are presented as the mean ± standard error (*n* = 6). *P < 0.05. **C**, **D** Representative western blots and quantification of Smad2/3, Smad4 and phosphorylated Smad2/3 (p-Smad2/3) in the hippocampus at day 3 after surgery. The data are presented as the mean ± standard error (*n* = 6). **P < 0.01. **E**–**H** Representative western blots and quantification of Smad2/3, Smad4 and p-Smad2/3 in the hippocampus at day 3 after the Smad7^−/−^ and wild-type mice underwent surgery. The data are presented as the mean ± standard error (*n* = 3). *P < 0.05, **P < 0.01. **I** General overview of the main highlights of this study. The expression of Smad7 increases significantly under anesthesia and surgery and inhibits the phosphorylation of Smad2/3, which amplifies inflammatory gene expression by decreasing the Smad2/3 and Smad4 complexes

The expression of TGF-β1 in the hippocampus was examined 3 days after unilateral nephrectomy, which demonstrated that the expression of TGF-β1 was significantly elevated in the hippocampus from the surgery group at both the mRNA and protein levels compared to the control group (Fig. 7A, B). Thereafter, the protein expression of Smad2/3, Smad4 and phosphorylated Smad2/3 in the hippocampus was determined through western blot assay 3 day postsurgery. The results revealed that the level of phosphorylated Smad2/3 but not Smad2/3 and Smad4 in the hippocampus from the surgery group was dramatically lower than that in the control group (Fig. 7C, D). To evaluate whether Smad7 is involved in the occurrence of cognitive impairment after anesthesia and surgery by regulating the TGF-β1-related signaling pathway, we detected the protein expression of Smad2/3, Smad4 and phosphorylated Smad2/3 in the hippocampus in wild-type mice and Smad7^−/−^ mice. No significant differences in the levels of Smad2/3 and Smad4 were observed in the hippocampus between the control and surgery groups (Fig. 7E–G). Interestingly, the phosphorylation of Smad2/3 was significantly upregulated in the hippocampus of Smad7^−/−^ mice compared to that of wild-type mice after surgery (Fig. 7E, H). Consequently, this study indicates that Samd7 plays a vital regulatory role in the development of cognitive dysfunction after anesthesia and surgery. As shown in Fig. 7I, under normal conditions, TGF-β receptor type I is activated by the binding of TGF-β to TGF-β receptor type II, which further promotes the phosphorylation of Smad2/3. After that, a complex that binds phosphorylated Smad2/3 and Smad4 develops and translocates into the nucleus, where the complex inhibits the transcription of inflammatory genes. However, in the development of cognitive impairment postoperation, the elevated Smad7 interacts with TGF-β receptor type I, thus inhibiting the phosphorylation of Smad2/3. As a consequence of defective TGF-β, the production of inflammatory factors in the hippocampus increased significantly after unilateral nephrectomy and eventually led to cognitive decline.

## Discussion

It is well-documented that elderly patients often suffer from cognitive dysfunction after anesthesia and surgery. Recently, the Nomenclature Consensus Working Group recommended the term “perioperative neurocognitive disorders (PND)” as an overarching term for cognitive dysfunction occurring in the preoperative or postoperative period, which included cognitive impairments diagnosed before operation, acute postoperative delirium, delayed neurocognitive recovery, and postoperative cognitive dysfunction (POCD) [8–13]. In the current study, we continued to use the term “POCD” due to our main focus on cognitive function during the postoperative period.

Preclinical studies have demonstrated memory decline in several POCD models, including tibial fracture surgery under isoflurane anesthesia [14], exploratory laparotomy under anesthesia [44], and excision of important organs [37]. Although various mechanisms have been proposed to be involved in the development of cognitive dysfunction after the anesthesia and surgery mentioned above, the underlying mechanism is still elusive. In the present work, unilateral nephrectomy under isoflurane anesthesia was performed to produce a postoperative dysfunction model in aged mice. Our results demonstrated that aged mice that underwent unilateral nephrectomy exhibited significant deficits in contextual fear memory in fear conditioning tests, while auditory cue fear memory exhibited a negligible decline. Since the aged mice did not alter total travel distance in the open field tests, further confirming that the memory impairments were unlikely to be attributed to the dysfunction in locomotor function and exploratory activities during the fear conditioning tests.

Increasing evidence has demonstrated that the disruption of Smad7 expression participates in a number of diseases, including inflammatory bowel disease [24], hepatic fibrosis [45], cardiac remodeling [46], cancer [47] and kidney disease [48]. However, little research has indicated the regulatory function of Smad7 in the development of cognitive impairment induced by anesthesia and surgery. Interestingly, we found that Smad7 was overexpressed in the hippocampus, a brain region closely associated with learning and memory function, after unilateral nephrectomy. Further studies revealed that the elevated hippocampal Smad7 was mainly located in neurons in the CA1 region. To determine whether the elevation of Smad7 was associated with cognitive decline postoperation, the expression of Smad7 in the hippocampal CA1 region was inhibited by shRNA–Smad7. The fear conditioning test showed that cognitive dysfunction postsurgery was attenuated significantly, suggesting that Smad7 overexpression in the hippocampus is heavily implicated in the occurrence of cognitive deficits after unilateral nephrectomy. In addition, Smad7 knockout mice in the hippocampal CA1 region were obtained by crossing Smad7^fl/fl^ conditional mutant mice and CaMKIIα-Cre line T29-1 transgenic mice. The Smd7^−/−^ mice exhibited significant improvement in learning and memory function after unilateral nephrectomy compared with the wild-type mice, further confirming that the elevation of Smad7 participated in cognitive impairment postoperation.

As demonstrated in previous reports, the overexpression of Smad7 was associated with sustained inflammation in IBDs and other chronic inflammatory disorders [24, 49]. In addition, accumulating studies have revealed a pivotal role for neuroinflammation in anesthesia- and surgery-induced learning and memory decline [15, 50]. The oversecretion of proinflammatory cytokines, including IL-1β, IL-6 and TNF-α, is associated with deficits in cognitive performance. In the present work, we found that the expression of these proinflammatory cytokines at the mRNA level was much higher in the hippocampus in aged mice that underwent unilateral nephrectomy than in those that did not undergo any operation. However, when Smad7 in the hippocampal CA1 region was knocked down by shRNA–Smad7 or knocked out through a transgene technique, the inflammatory responses in the hippocampus decreased significantly. In addition, chemokines, including CCL2, CXCL1 and CXCL2, exhibit vital regulatory effects in the development of inflammation [51–53]. We found that anesthesia increased the mRNA levels of CCL2, CXCL1 and CXCL2 in the hippocampus, which was reversed by Smad7 knockdown or knockout. These results indicated that the unilateral nephrectomy-induced overexpression of Smad7 might take part in neuroinflammatory reactions. However, the molecular mechanisms that enhance neuroinflammatory responses postoperation remain poorly understood.

Smad7 has been confirmed to be implicated in the pathogenesis and treatment of inflammatory diseases as a negative regulator of TGF-β [24]. The biological activities of the multifunctional signaling protein TGF-β are mediated by both type I and type II TGF-β receptors. The transduction of TGF-β signal was initiated through binding of TGF-β to TGF-β receptor type II. Then, TGF-β type I was activated and led to the phosphorylation of Smad2 and Smad3. Phosphorylated Smad2/3 forms a heteromeric complex with Smad4 and inhibits the transcription of inflammatory genes after translocating into the nucleus. This role in regulating inflammatory responses has been proven in several pathological processes, such as kidney diseases and IBDs [27, 29, 30, 54]. In the hippocampus of the mice that underwent unilateral nephrectomy, we examined upregulated TGF-β, which stimulates the transcription of Smad7 consistent with the overexpression of Smad7 in this study. Extensive evidence has indicated that elevated Smad7 blocks Smad2/3 phosphorylation by interacting with TGF-β receptor type I and further preventing signal transduction of TGF-β, which increases inflammatory gene expression [29]. Interestingly, the phosphorylation of Smad2/3 was significantly reduced in the hippocampus from the surgery group compared with the control group, which was in accordance with the enhanced inflammation in the hippocampus after unilateral nephrectomy. However, when the expression of Smad7 in the hippocampal CA1 region was downregulated, increased levels of phosphorylated Smad2/3 were observed in the hippocampus, which recovered the suppressive effect of TGF-β on the transcription of inflammatory factors. Importantly, other researchers demonstrated that when Smad7 was silenced by an antisense Smad7 oligonucleotide in patients with IBDs and other mouse models of CNS autoimmunity, inflammatory reactions were attenuated significantly [55]. Meanwhile, this Smad7 inhibitor Mongersen exhibited modest success in a single-arm open-label study in a phase II trial of UC as detected by clinical response and endoscopic remission (ClinicalTrails.gov: NCTO2601300) [24]. In the present work, the results demonstrated that the specific downregulation of Smad7 in the hippocampal CA1 region indeed protected mice from neuroinflammation after unilateral nephrectomy, which further improved the cognitive decline induced by anesthesia surgery. Consequently, Smad7 might be a promising therapeutic target in the regulation of cognitive dysfunction by suppressing inflammatory responses.

In addition to the enhancement of neuroinflammation, our results further demonstrate that apoptosis in the hippocampus increased significantly. Apoptosis was inhibited through the knockdown or knockout of Smad7. However, the underlying mechanism of TGF-β- and Smad7-induced apoptosis in postoperation cognitive decline is unclear. Increasing evidence indicates that Smad7 plays a vital role in the regulation of TGF-β-induced apoptosis, with the exception of TGF-β signaling [32, 56]. Another report demonstrated that Smad7 overexpression but not regulatory Smad2/3 could enhance apoptosis in mesangial cells. In addition, antisense oligonucleotides against Smad7 reversed TGF-β-triggered apoptosis in mesangial cells [31]. Interestingly, our results also demonstrated the role of Smad7 in neuronal apoptosis induced by TGF-β. Thus, Smad7 might be involved in TGF-β-induced apoptosis by interacting with other proapoptotic molecules that have been proven to be closely associated with TGF-β-induced apoptosis, including the activation of p38 or NF-κB [32]. However, the relationship between these proapoptotic factors and Smad7 has not been clarified in our research, although we have revealed that the elevation of Smad7 takes part in the development of apoptosis.

Our study certainly has some limitations. We mainly evaluated the cognitive function of the mice through the fear conditioning test in the present work, which seems to be insufficient. Meanwhile, we used a forward conditioning procedure to investigate the animal’s ability to acquire and retrieve an association between conditioned stimulus and unconditioned stimulus. We failed to use the backward conditioning procedure as a control to indirectly retrieve and manipulate the contextual fear engram in mice. Increasing evidence has demonstrated that anesthetic drugs, such as isoflurane and propofol, play vital roles in regulating molecular pathology changes in the central nervous system and neurocognitive behaviors. It is necessary to design a group of mice solely undergoing isoflurane anesthesia as a control to exclude drug interference in cognitive deficits postoperation [38, 39, 57]. Therefore, more experiments are still needed to confirm the effect of Smad7 in regulating the cognitive decline induced by anesthesia surgery in the future, which would provide an important theoretical basis for the molecular pathological mechanism and clinical intervention of cognitive impairment caused by anesthesia surgery.

In conclusion, our study demonstrated that the overexpression of Smad7 in the hippocampus contributed to unilateral nephrectomy-induced cognitive dysfunction by increasing neuroinflammatory responses and apoptosis. The elevated expression of Smad7 in the hippocampus was heavily implicated in the overexpression of TGF-β in the hippocampus after anesthesia operation. Afterwards, the upregulated Smad7 inhibited the phosphorylation of Smad2/3 by interacting with TGF-β receptor type I, which blocked the signal transduction of TGF-β. As a consequence of defective TGF-β signaling, the inflammatory responses were enhanced significantly and further led to cognitive dysfunction. Meanwhile, the increased expression of Smad7 and TGF-β simultaneously induced apoptosis in the hippocampus, which also had an adverse function in the development of cognitive decline after unilateral nephrectomy. Importantly, neuroinflammation and apoptosis were reduced in the hippocampus when the expression of Smad7 was inhibited by shRNA–Smad7 and the transgene technique, which attenuated learning and memory impairment induced by anesthesia surgery. The current work provides evidence that Smad7 could be a promising therapeutic target for the clinical treatment of cognitive dysfunction induced by anesthesia and surgery.

## Supplementary Information

**Additional file 1: Figure S1**. A-B. Quantitative analysis of Smad7 expression in hippocampal (A) CA1 and (B) CA2 regions according to immunofluorescent images by ImageJ software. The data are presented as the mean ± standard error (n = 3). *P < 0.05. **Figure S2.** A-B. Representative western blots and quantification of Smad7 in the (A) prefrontal cortex and (B) amygdala at day 3 after surgery. **Figure S3**. Gene identification of Smad7 knockout mice by direct PCR analysis of tail DNA. A. Identification of Smad7 gene. B. Identification of the Cre gene. Line ① represents the gene of wild-type mice. Line ② represents the blank control. Lines ③-⑤ represent the genes of the Smad7^-/-^ mice without any process. Lines ⑥-⑦ represent the gene of the Smad7^-/-^ mice after surgery. **Figure S4**. Full and unprocessed western blot images corresponding to Figure 2B, the square refers to the blots cited in the main article. **Figure S5**. Full and unprocessed western blot images corresponding to Figure 3B, the square refers to the blots cited in the main article. **Figure S6**. Full and unprocessed western blot images corresponding to Figure 4B, the square refers to the blots cited in the main article. **Figure S7**. Full and unprocessed western blot images corresponding to Figure 6A, the square refers to the blots cited in the main article. **Figure S8**. Full and unprocessed western blot images corresponding to Figure 6D, the square refers to the blots cited in the main article. **Figure S9**. Full and unprocessed western blot images corresponding to Figure 7B, the square refers to the blots cited in the main article. **Figure S10**. Full and unprocessed western blot images corresponding to Figure 7C, the square refers to the blots cited in the main article. **Figure S11**. Full and unprocessed western blot images corresponding to Figure 7E.

## Data Availability

All data generated or analyzed during this study are included in this published article and its Additional files.
