# Supplementary material for: Smad7 in the hippocampus contributes to memory impairment in aged mice after anesthesia and surgery
Source: J Neuroinflammation. 2023 Jul 28;20:175. doi: 10.1186/s12974-023-02849-z (PMC10375636; doi:10.1186/s12974-023-02849-z)
Supplement: Supplementary file 1 — Additional file 1: Figure S1. A-B. Quantitative analysis of Smad7 expression in hippocampal (A) CA1 and (B) CA2 regions according to immunofluorescent images by ImageJ software. The data are presented as the mean ± standard error (n = 3). *P < 0.05. Figure S2. A-B. Representative western blots and quantification of Smad7 in the (A) prefrontal cortex and (B) amygdala at day 3 after surgery. Figure S3. Gene identification of Smad7 knockout mice by direct PCR analysis of tail DNA. A. Identification of Smad7 gene. B. Identification of the Cre gene. Line ① represents the gene of wild-type mice. Line ② represents the blank control. Lines ③-⑤ represent the genes of the Smad7-/- mice without any process. Lines ⑥-⑦ represent the gene of the Smad7-/- mice after surgery. Figure S4. Full and unprocessed western blot images corresponding to Figure 2B, the square refers to the blots cited in the main article. Figure S5. Full and unprocessed western blot images corresponding to Figure 3B, the square refers to the blots cited in the main article. Figure S6. Full and unprocessed western blot images corresponding to Figure 4B, the square refers to the blots cited in the main article. Figure S7. Full and unprocessed western blot images corresponding to Figure 6A, the square refers to the blots cited in the main article. Figure S8. Full and unprocessed western blot images corresponding to Figure 6D, the square refers to the blots cited in the main article. Figure S9. Full and unprocessed western blot images corresponding to Figure 7B, the square refers to the blots cited in the main article. Figure S10. Full and unprocessed western blot images corresponding to Figure 7C, the square refers to the blots cited in the main article. Figure S11. Full and unprocessed western blot images corresponding to Figure 7E. [file 12974_2023_2849_MOESM1_ESM.docx]

**Additional information for**

**Smad7 in the hippocampus contributes to memory impairment in aged mice after anesthesia and surgery**

Changliang Liu^1,2,#^, Jiahui Wu^1,2,#^, Ming Li^1^, Rui Gao^1^, Xueying Zhang^1^, Shixin Ye-Lehmann^3^, Jiangning Song^4^, Tao Zhu^1,2,*^, Chan Chen^1,2,*^

^1^Department of Anesthesiology, West China Hospital, Sichuan University, Chengdu, China.

^2^Laboratory of Anesthesia and Critical Care Medicine, National-Local Joint Engineering Research Center of Translational Medicine of Anesthesiology. West China Hospital, Sichuan University, Chengdu, China.

^3^Diseases and Hormones of the Nervous System University of Paris-Scalay Bicêtre Hosptial Bât. Grégory Pincus 80 Rue du Gal Leclerc 94276 Le Kremlin Bicêtre CEDEX, Paris, France.

^4^Monash Biomedicine Discovery Institute and Department of Biochemistry and Molecular Biology, Monash University, Melbourne, VIC, Australia.

*Correspondence to: Chan Chen, email: chenchan@scu.edu.cn or xychenchan@gmail.com; Tao Zhu, email: xwtao.zhu@foxmail.com

^#^These authors contributed equally to this paper.


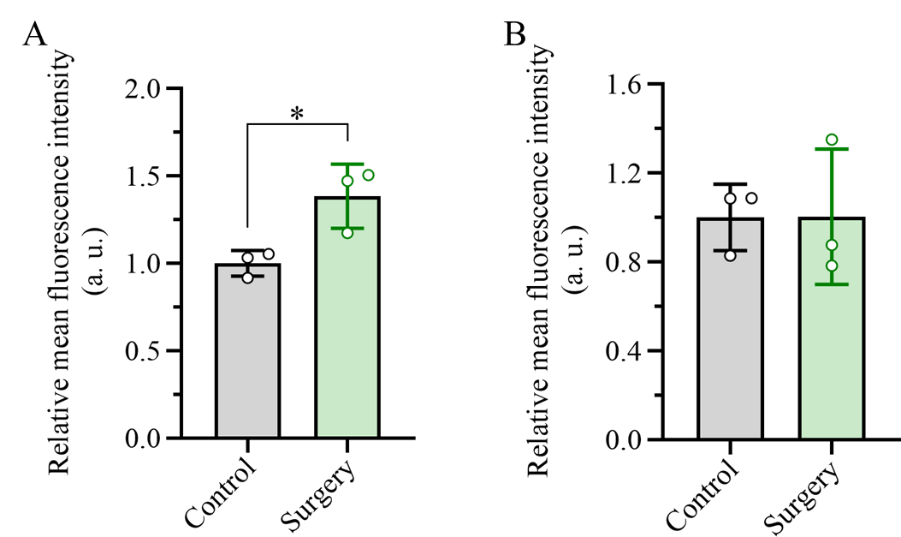


**Figure S1**. A-B. Quantitative analysis of Smad7 expression in hippocampal (A) CA1 and (B) CA2 regions according to immunofluorescent images by ImageJ software. The data are presented as the mean ± standard error (n = 3). **P* < 0.05.


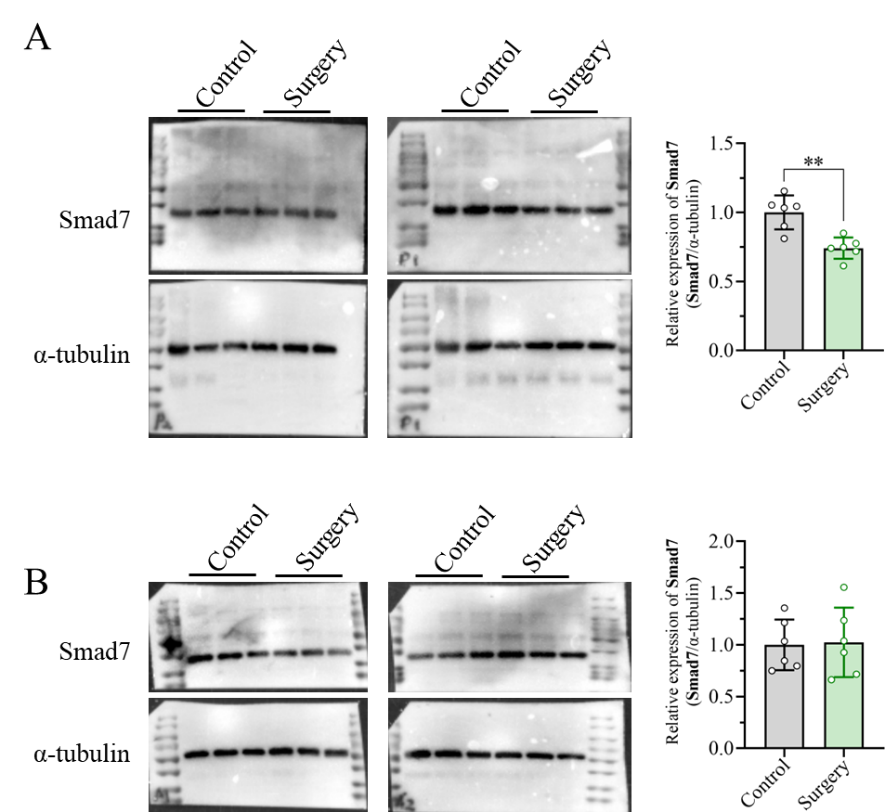


**Figure S2.** A-B. Representative western blots and quantification of Smad7 in the ()A prefrontal cortex and (B) amygdala at day 3 after surgery.


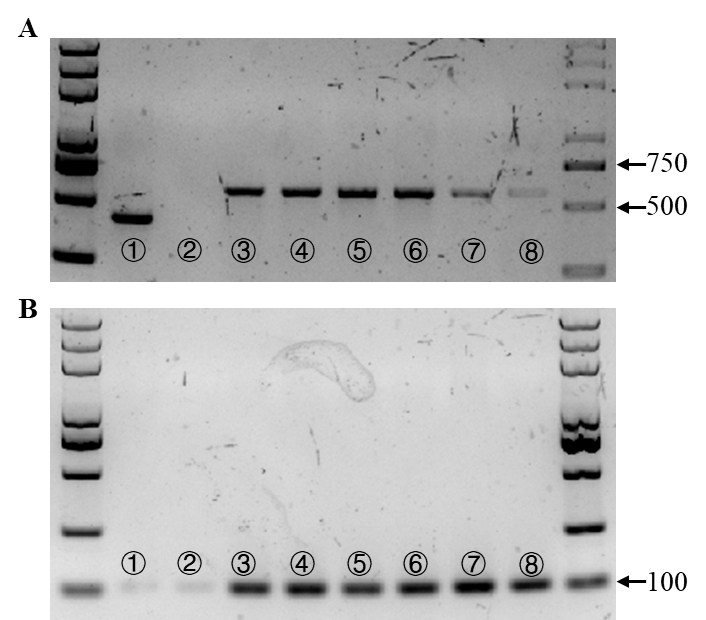


**Figure S3**. Gene identification of Smad7 knockout mice by direct PCR analysis of tail DNA. A. Identification of Smad7 gene. B. Identification of the Cre gene. Line ① represents the gene of wild-type mice. Line ② represents the blank control. Lines ③-⑤ represent the genes of the Smad7^-/-^ mice without any process. Lines ⑥-⑦ represent the gene of the Smad7^-/-^ mice after surgery.


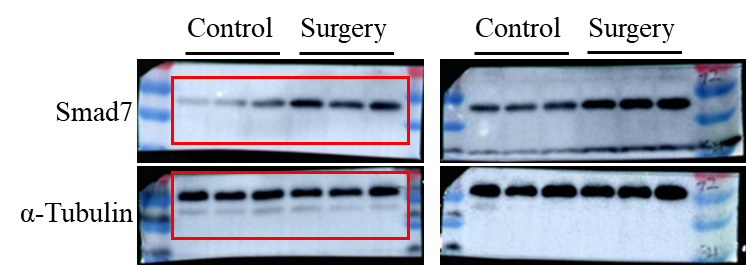


**Figure S4**. Full and unprocessed western blot images corresponding to Figure 2B, the square refers to the blots cited in the main article.


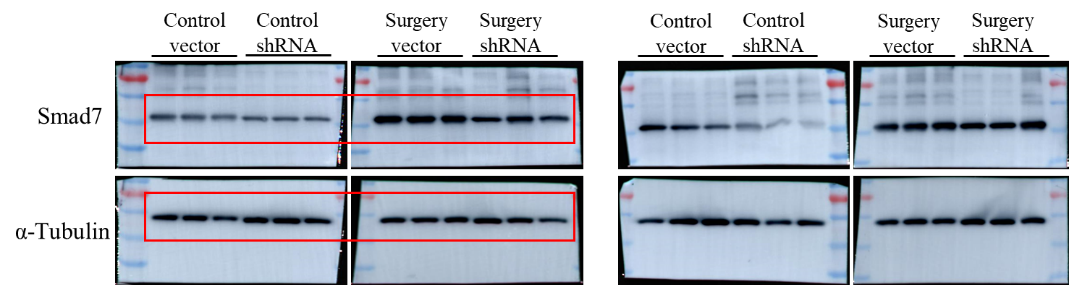


**Figure S5**. Full and unprocessed western blot images corresponding to Figure 3B, the square refers to the blots cited in the main article.


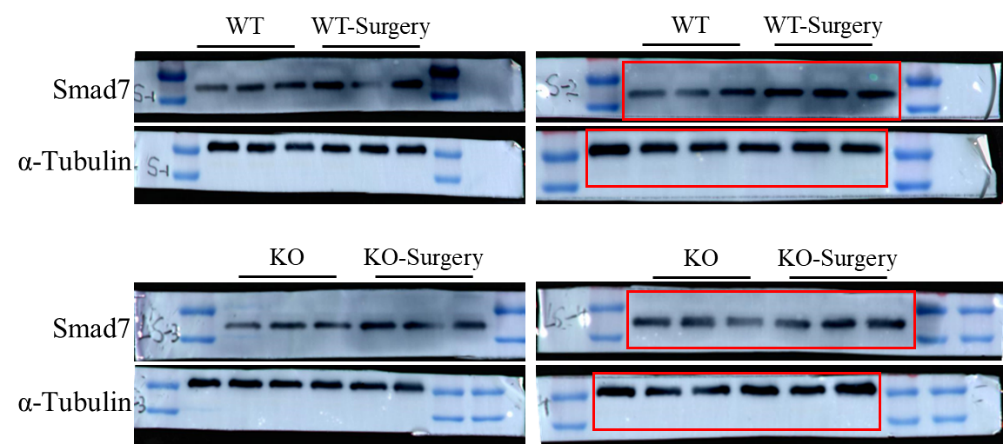


**Figure S6**. Full and unprocessed western blot images corresponding to Figure 4B, the square refers to the blots cited in the main article.


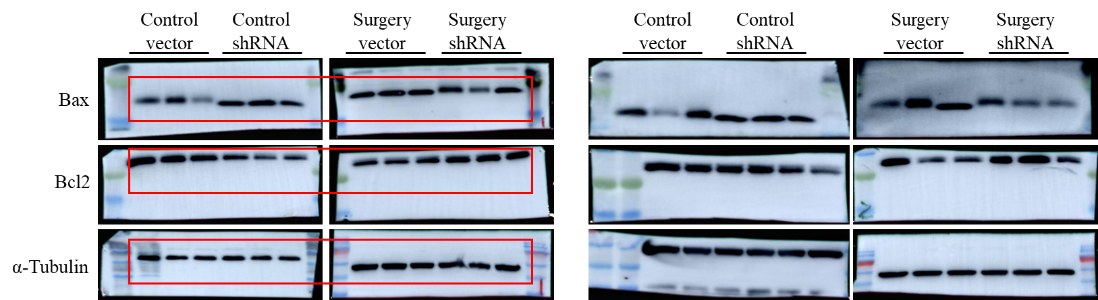


**Figure S7**. Full and unprocessed western blot images corresponding to Figure 6A, the square refers to the blots cited in the main article.


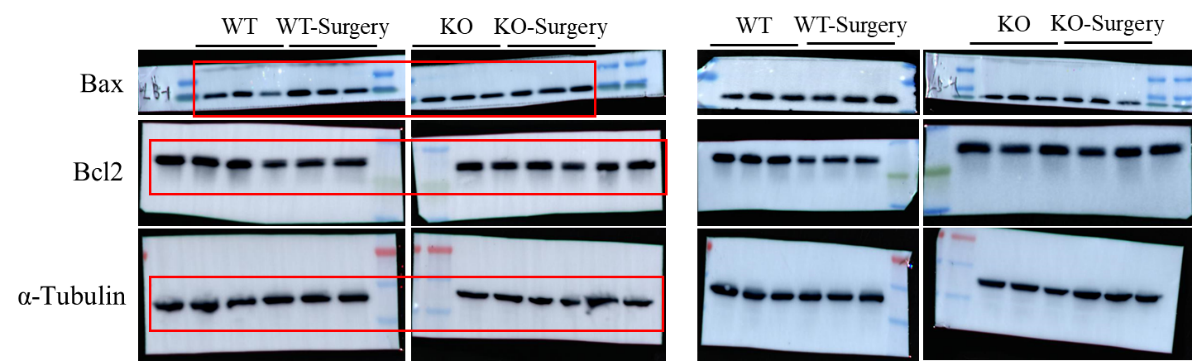


**Figure S8**. Full and unprocessed western blot images corresponding to Figure 6D, the square refers to the blots cited in the main article.


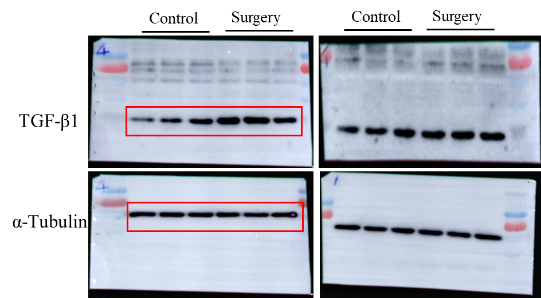


**Figure S9**. Full and unprocessed western blot images corresponding to Figure 7B, the square refers to the blots cited in the main article.


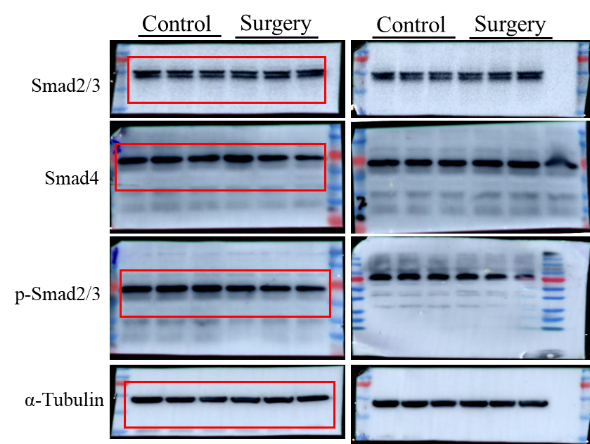


**Figure S10**. Full and unprocessed western blot images corresponding to Figure 7C, the square refers to the blots cited in the main article.


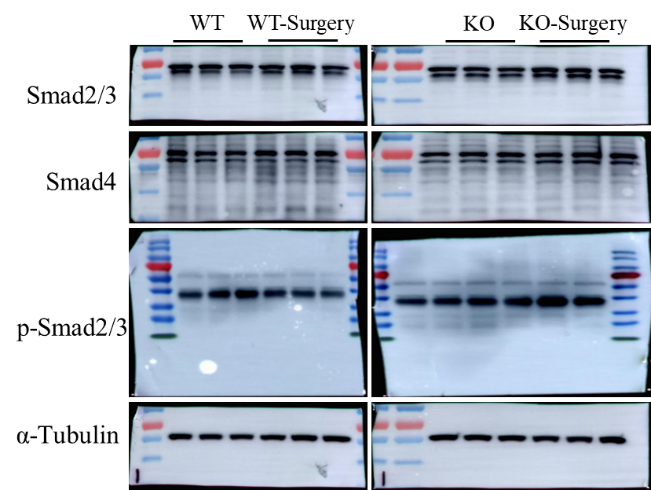


**Figure S11**. Full and unprocessed western blot images corresponding to Figure 7E.
